# The mTOR inhibition in concurrence with ERK1/2 activation is involved in excessive autophagy induced by glycyrrhizin in hepatocellular carcinoma

**DOI:** 10.1002/cam4.1127

**Published:** 2017-07-03

**Authors:** Xuan Zhang, Hua Yang, Shuqiang Yue, Guangbin He, Shibin Qu, Zhuochao Zhang, Ben Ma, Rui Ding, Wei Peng, Hongtao Zhang, Zhaoxu Yang, Kefeng Dou, Kaishan Tao, Xiao Li

**Affiliations:** ^1^ Department of Hepatobiliary Surgery Xijing Hospital The Fourth Military Medical University Xi'an Shaanxi China; ^2^ Department of Geriatrics Xi'an No. 1 Hospital Xi'an Shaanxi China; ^3^ Department of Oncological Surgery Xijing Hospital The Fourth Military Medical University Xi'an Shaanxi China; ^4^ Department of Ultrasound Diagnosis Xijing Hospital The Fourth Military Medical University Xi'an Shaanxi China

**Keywords:** Autophagy, ERK1/2, glycyrrhizin, hepatocellular carcinoma, mTOR

## Abstract

Autophagy is a life phenomenon in which autophagosomes remove damaged or aging organelles and long‐lived circulating proteins to maintain the cell's stability. However, disorders of excessive autophagy are a response of cancer cells to a variety of anticancer treatments which lead to cancer cell death. The Akt/mammalian target of rapamycin (mTOR) and the extracellular signal‐regulated kinase 1/2 (ERK1/2) pathways are both involved in nutrient‐induced autophagic phenomenon and exhibit vital relevance to oncogenesis in various cancer cell types, including hepatocellular carcinoma (HCC). However, the influence of autophagy for cancer cell death remains controversial and few scientists have investigated the variation of these two signaling pathways in cancer cell autophagic phenomenon induced by anticancer treatment simultaneously. Here, we explored the anticancer efficacy and mechanisms of glycyrrhizin (GL), a bioactive compound of licorice with little toxicity in normal cells. It is interesting that inhibition of Akt/mTOR signaling in concurrence with enhanced ERK1/2 activity exists in GL‐induced autophagy and cytotoxicity in HepG2 and MHCC97‐H hepatocellular carcinoma cells. These results imply that the GL‐related anticancer ability might correlate with the induction of autophagy. The influence of induced autophagic phenomenon on cell viability might depend on the severity of autophagy and be pathway specific. In the subsequent subcutaneous xenograft experiment in vivo with MHCC97‐H cells, GL obviously exhibited its inhibitory efficacy in tumor growth via inducing excess autophagy in MHCC97‐H cells (*P* < 0.05). Our data prompt that GL possesses a property of excess autophagic phenomenon induction in HCC and exerts high anticancer efficacy in vitro and in vivo. This warrants further investigation toward possible clinical applications in patients with HCC.

## Introduction

In recent years, hepatocellular carcinoma (HCC) has been categorized as the sixth most common malignant tumor worldwide 1. Despite dramatic advances in cancer therapy, such as the combination of surgery, transhepatic artery chemoembolization, radiofrequency ablation, chemotherapy, and radiotherapy, the overall prognosis of HCC remains dismal, and the 5‐year survival rate of patients with advanced HCC remains less than 10% 2, 3, 4, which is due to high recurrence and metastatic rates 2. Therefore, there is an urgent demand to develop new therapeutic strategies.

Glycyrrhizin (GL) is the bioactive component of licorice and it has recently been used as an herbal medicine due to its antiviral 5, anticancer 6, and anti‐inflammatory properties 7. However, very limited researches have focused on the anticancer efficacy of GL in human hepatocellular carcinoma (HCC), and the detailed molecular mechanism refers to the tumor inhibition of GL in HCC is not yet fully elucidated. Various molecules and signal pathways might contribute to the anticancer efficacy of GL. GL could reduce the expression of NF‐*κ*B (p65) and induce apoptosis in several cancer cell lines including human glioblastoma 6, stomach cancer, promyelocytic leukemia 8, 9, and prostate cancer 10. GL had been proved in its ability of decreasing antigenic activities in endothelial cells via reducing the production of reactive oxygen species and activating the ERK pathway 11. GL could also activate caspase‐ and mitochondria‐dependent apoptosis pathways 12 and block Akt/mTOR/STAT3 signaling 13 to restrain the growth of leukemia cell lines. In addition, GL depressed thromboxane synthase to restrict the growth of lung adenocarcinoma cells 14. Furthermore, GL ameliorated radiation‐induced tumorigenesis via downregulation of thymine dimer, proliferative cell nuclear antigen, apoptosis, and transcription factor NF‐*κ*B 15.

Autophagy is an essentially protein degradation system of the cell lysosomes 16, and it has garnered attention in the field of cancer research because it is designated “programmed cell death type II” 17 and participates in the oncogenesis and development of many types of cancer diseases. For example, autophagy potentiated the anticancer effects of the histone deacetylase inhibitors in hepatocellular carcinoma 18. Rapamycin exerted its antitumor effect on malignant glioma cells by inducing autophagy, and a disruption of the PI3K/Akt signaling pathway could greatly enhance the effectiveness of mTOR inhibitors 19, 20. Furthermore, oxidative stress induced autophagic cell death independent of apoptosis in the transformed cell line HEK293 and cancer cell lines U87 and HeLa 21.

Both mTOR and ERK1/2 participate in the regulation of autophagy. Amino acids and ATP could activate the mTOR/p70S6K pathway and negatively regulate autophagic phenomenon 22. Moreover, the mTOR pathway had upstream regulators: PTEN and Akt. While PTEN exerted its ability of autophagy induction, Akt exhibited an opposite role 23. Meanwhile, autophagy could also be restrained by Raf‐1/MEK1/2/ERK1/2 pathway which exhibited a negative relevance to amino acids. ERK phosphorylated G*α*‐interacting protein which further accelerated the rate of GTP hydrolysis by the G*α*_i3_ protein and then resulted in induction of autophagy 24, 25. Although these two important signal pathways have an established connection with autophagy, their roles in autophagy are not yet fully confirmed and illustrated in cancer.

## Materials and Methods

### Cell culture and drug

Human HCC cell lines HepG2 and MHCC97‐H were obtained from the Cell Bank of the Chinese Academy of Sciences (Shanghai, China). Cells were routinely cultured in Dulbecco's modified Eagle's medium (DMEM, HyColone, Logan, UT) supplemented with 10% fetal bovine serum (FBS, Invitrogen, Carlsbad, CA) and 1% penicillin–streptomycin at 37°C and 5% CO_2_ in a humidified incubator. Dry GL powder (purity>95.0%, from Sigma‐Aldrich, MO) was prepared in a 10 mmol/L stock solution with culture medium and was then diluted into required concentrations with culture medium for need. 3‐Methyladenine (3‐MA), a *β* type PI3K inhibitor which can combine with Vps34 to block the formation of autophagosome, and chloroquine, a proteolysis inhibitor, were purchased from Sigma‐Aldrich. Atg7 siRNA was used to silence autophagy‐essential gene *Atg7* to verify the role of 3‐MA (Life Technologies, CA).

### Determination of cell viability

Cells were seeded into 96‐well plates at 3 × 10^3^ cells per well and then administered with 0, 1, 2, and 4 mmol/L GL for 24, 48, and 72 h. Cell viability was detected using a CCK‐8 assay kit (Beyotime, Jiangsu, China) according to the manufacturer's instructions. Cell viability was determined by measuring NADH production, resulting from dehydrogenase activity in viable cells. Briefly, each well was added with 10 *μ*L of CCK‐8 solution and incubated at 37°C for 2 h. Thereafter, the optical density (OD) for each well was measured at 450 nm using a microplate reader (Bio‐Rad Model 680, USA). All experiments were repeated three times, and the mean value was calculated. The cell survival equation: cell survival rate = (OD experiment − OD blank)/(OD control − OD blank) × 100%. To verify the role of excessive autophagy in glycyrrhizin‐mediated cell death in vitro, autophagy was inhibited by 3‐MA (2 mmol/L) or Atg7 siRNA (50 nmol/L) before GL was administered. For autophagic flux, cells were treated with 10 *μ*mol/L chloroquine.

### siRNA transfection

Atg7 siRNA was used to inhibit Atg7 expression. Transfection was conducted using Lipofectamine RNAi reagent (Life Technologies) according to the manufacturer's instructions. A scrambled siRNA was transfected as the negative control. After transfection for 48 h, HCC cells were treated with GL for further analysis.

### Plate colony‐forming assay

Cells in logarithmic growth phase were harvested and resuspended into a single cell suspension with DMEM supplemented with 10% FBS and then seeded in a six‐well plate at a density of 300 cells per well in triplicate. They were cultured in growth medium supplemented with GL (0, 1, or 2 mmol/L) for 18 days. Then, the colonies were fixed in 95% ethanol and stained with a 4 g/L crystal violet solution, and colony formation was photographed and counted under a 100× magnification. Colonies containing over 50 cells were counted.

### Cell migration assays

Cell migration was detected using Transwell chambers (8 *μ*m pore size; Millipore) without Matrigel matrix. In brief, cells in logarithmic growth phase were harvested and administered to GL in serum‐free medium at different concentrations (0, 1, or 2 mmol/L), 600 *μ*L of complete medium was added to the bottom chamber and 200 *μ*L of the cell suspension (containing 4 × 10^4^ cells) was placed in the upper chamber. After 12 h, the cells on the top surface of the membrane were mechanically removed using a cotton swab, and the cells on the bottom surface of the membrane were fixed in 95% ethanol and stained with a 4 g/L crystal violet solution. Cells adhering to the bottom surface of the membrane were counted in five randomly selected areas under a 100× microscope field. Each experiment was repeated three times.

### Acridine orange immunofluorescent staining

The formation of acid vesicular organelles (AVOs) was assessed by acridine orange (AO) staining (Sigma Chemical Co., St. Louis, USA) and then observed with fluorescence microscopy. After treatment with glycyrrhizin for 12 h, cells were stained with 20 *μ*g/mL AO in medium and incubated in the dark for 15 min at 37°C. Then, cells were washed with PBS four times, and an inverted fluorescence microscope (Olympus, Japan) was used to obtain fluorescent micrographs.

### Western blot analysis

Cells or tumor tissue extracts were digested in RIPA lysis buffer (Beyotime, Shanghai, China) containing protease and phosphatase inhibitors (Thermo Scientific, Rockford, IL). The proteins were quantified using a BCA protein assay kit (Thermo Scientific) according to the manufacturer's instructions. The total protein extracts were separated by 10% SDS‐PAGE and transferred to polyvinylidene difluoride membranes. The membranes were incubated with primary antibodies, including anti‐LC3B (Abcam, Cambridge, UK), anti‐ERK1/2 (Cell Signaling Technology CST, Danvers, MA), anti‐p‐ERK1/2(CST), anti‐mTOR (CST), anti‐p‐mTOR (CST), anti‐p85 (Abcam), anti‐p‐p85 (Abcam), anti‐Akt (CST), anti‐p‐Akt (CST), anti‐p70S6K (CST), anti‐p‐p70S6K (CST), anti‐p62 (CST), anti‐Craf (Abcam), anti‐p‐Craf (Abcam), anti‐Atg7 (CST), anti‐GAPDH and *β*‐actin (Abmart, China) overnight at 4°C. Then, the membranes were incubated with horseradish peroxidase (HRP)‐conjugated secondary antibodies (Abcam) for 1 h at room temperature, and the protein bands were detected with a ChemiDoc^™^ XRS+ and Image Lab TM software (Bio‐Rad, Hercules, CA).

### Subcutaneous xenografts in nude mice

Nude mice (BALB/C nu/nu, 5‐week‐old, female) were purchased from HFK Bioscience Ltd. (Beijing, China) and maintained under specific pathogen‐free conditions in the laboratory animal center of Fourth Military Medical University (Xi'an, China). All experiments were performed in accordance with the Guide for the Care and Use of Laboratory Animals and were approved by The Research Animal Care and Use Committee of Fourth Military Medical University.

For the tumorigenesis assay, MHCC97‐H cells (3 × 10^6^) were suspended in a 100 *μ*L mixture of equal volumes of medium and Matrigel and were implanted subcutaneously into the left flank of nude mice (n = 3 mice per group). When the tumors reached a volume of approximately 50–70 mm^3^, the mice were then randomly divided into two groups. The treatment group received a peritumoral administration of glycyrrhizin (100 mg/kg body weight in 80 *μ*L olive oil, *n* = 3), whereas the vehicle control group received olive oil alone (*n* = 3). These treatments were carried out once daily for 40 days. The tumor volumes and the body weight of the mice were measured twice per week. The tumor volumes (mm^3^) were calculated with the following formula: tumor volume = (length × width^2^)/2. At the end of the experiment, the mice were sacrificed, and the tumors were harvested and weighed. The dissected tumors were frozen in liquid nitrogen for western blot analysis or fixed in formalin and embedded in paraffin for immunohistochemistry.

### Immunohistochemistry

The tissue samples were fixed in formalin, embedded in paraffin, and sectioned to a 4‐*μ*m thickness. The sections were deparaffinized, hydrated, and boiled in 10 mmol/L citrate buffer (pH 6.0) for antigen retrieval. Endogenous peroxidases were inactivated with 3% H_2_O_2_. After the sections were blocked in goat serum, the sections were incubated with primary antibody (LC3B, Abcam, Cambridge, UK; Ki‐67, Boster, Wuhan, China) overnight at 4°C, incubated with biotinylated secondary antibody at room temperature for 1 h, and visualized with diaminobenzidine (DAB kit, ZSGB‐BIO, China). Hematoxylin was used to counterstain the nuclei.

### Statistical analysis

All data are presented mean ± SD. Intragroup comparisons were made by employing Student's *t*‐test or an analysis of variance. All statistical analyses were performed using SPSS version 17.0 (IBM, Chicago, IL), and all figures were generated using GraphPad Prism 5.01 (GraphPad Software, La Jolla, CA). In all cases, a *P* < 0.05 was considered to be statistically significant.

## Results

### Cytotoxicity of glycyrrhizin in HCC cells

**Figure 1:**
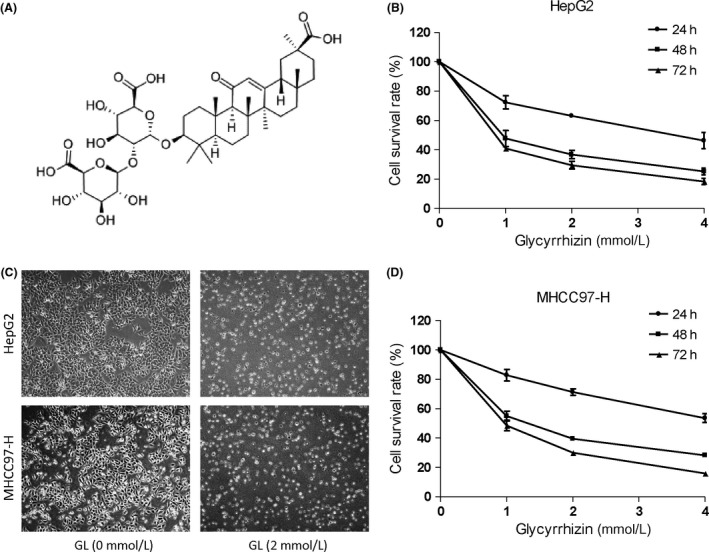
Glycyrrhizin inhibits HepG2 and MHCC97‐H cell survival. (A) Structure of glycyrrhizin. (B) and (D) HepG2 and MHCC97‐H cells were exposed to the indicated concentrations of glycyrrhizin (0, 1, 2, or 4 mmol/L) for 24, 48, or 72 h. Cell viability was determined by a CCK‐8 solution cell viability assay. (C) Representative images of cell morphology in HepG2 and MHCC97‐H cells were taken after a 72‐h treatment with or without 2 mmol/L glycyrrhizin. Curves, mean (*n* = 3, in triplicate).

To explore whether GL has direct antitumor activity, we tested GL‐induced cytotoxicity in two types of HCC cell lines, including well‐differentiated HepG2 and poorly differentiated MHCC97‐H cells. Dose‐dependent and time‐dependent studies were performed in the human HCC cell lines described above. Relatively viable cells were tested by a CCK‐8 assay. As shown in Figure 1B and D, cells administered with GL had a dramatically reduced viability and exhibited obvious inhibition of cell survival in a dose‐ and time‐dependent manner. The EC50 value for growth inhibition was calculated in GL‐treated HCC cells. GL exerted approximately 50% inhibition at 1 mmol/L after 48 h treatment. Representative images of cell morphology showed that GL significantly killed HepG2 and MHCC97‐H cells (Fig. 1C). Collectively, these data indicate that GL has potent antitumor effects on human hepatocellular carcinoma cells.

### Inhibition of glycyrrhizin on colony‐forming capacity and migration in HCC cells

**Figure 2:**
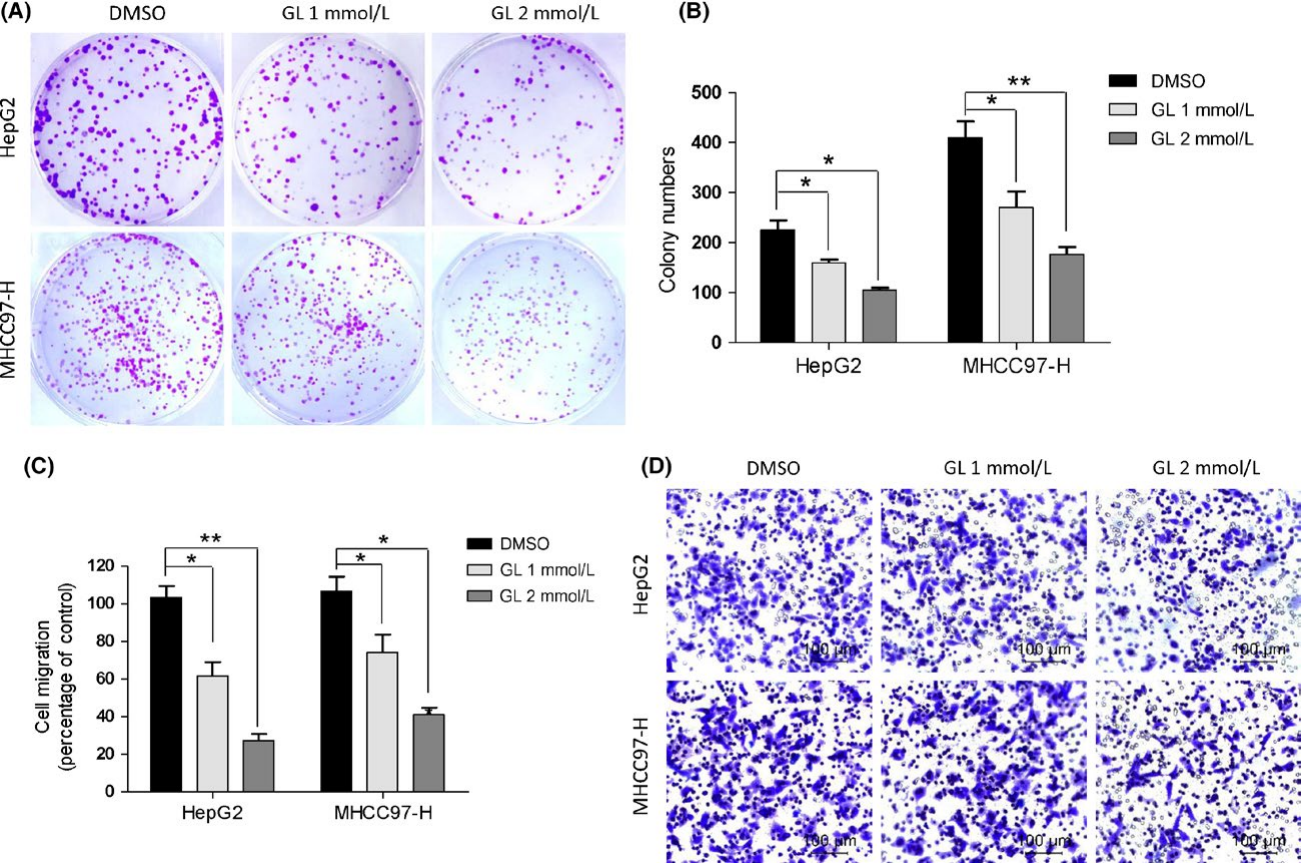
Glycyrrhizin inhibits HCC cells colony‐forming potential and migration. (A) Colonies are shown after seeding 300 HepG2 and MHCC97‐H cells/plate. (B) Graph exhibits the efficiencies expressed as colony‐forming capacity. Values represent the average number of formed colonies. (C) and (D) Glycyrrhizin inhibited HepG2 and MHCC97‐H migration in a transwell assay without a Matrigel matrix. The bottom chambers of the transwells were filled with 600 *μ*L of complete DMEM, whereas the top chambers were seeded with 4 × 10^4^ HepG2 and MHCC97‐H in serum‐free DMEM and treated with different concentrations of glycyrrhizin for 12 h. Cell migration through the membrane was stained and quantified. Columns, mean (*n* = 3, in triplicate); bars, SD. *P < 0.05; **P < 0.01.

A colony formation assay was carried out to investigate the inhibitory efficiency of GL on HCC cell proliferation. The results showed that HepG2 and MHCC97‐H cells exposed to different concentration of GL had a significant reduction in colony counts compared to the controls (Fig. 2A and B). Moreover, we explored the impact of GL on the migration of HCC cells in Transwell assays without Matrigel matrix. We observed that GL markedly suppressed the migration of HepG2 and MHCC97‐H cells in a dose‐dependent manner. The migration rates of HepG2 cells treated with 1 and 2 mmol/L GL were 61.67% and 27.33%, respectively. A similar phenomenon was observed in MHCC97‐H cells (Fig. 2C and D).

### Glycyrrhizin evokes excessive autophagy in HCC cells

**Figure 3:**
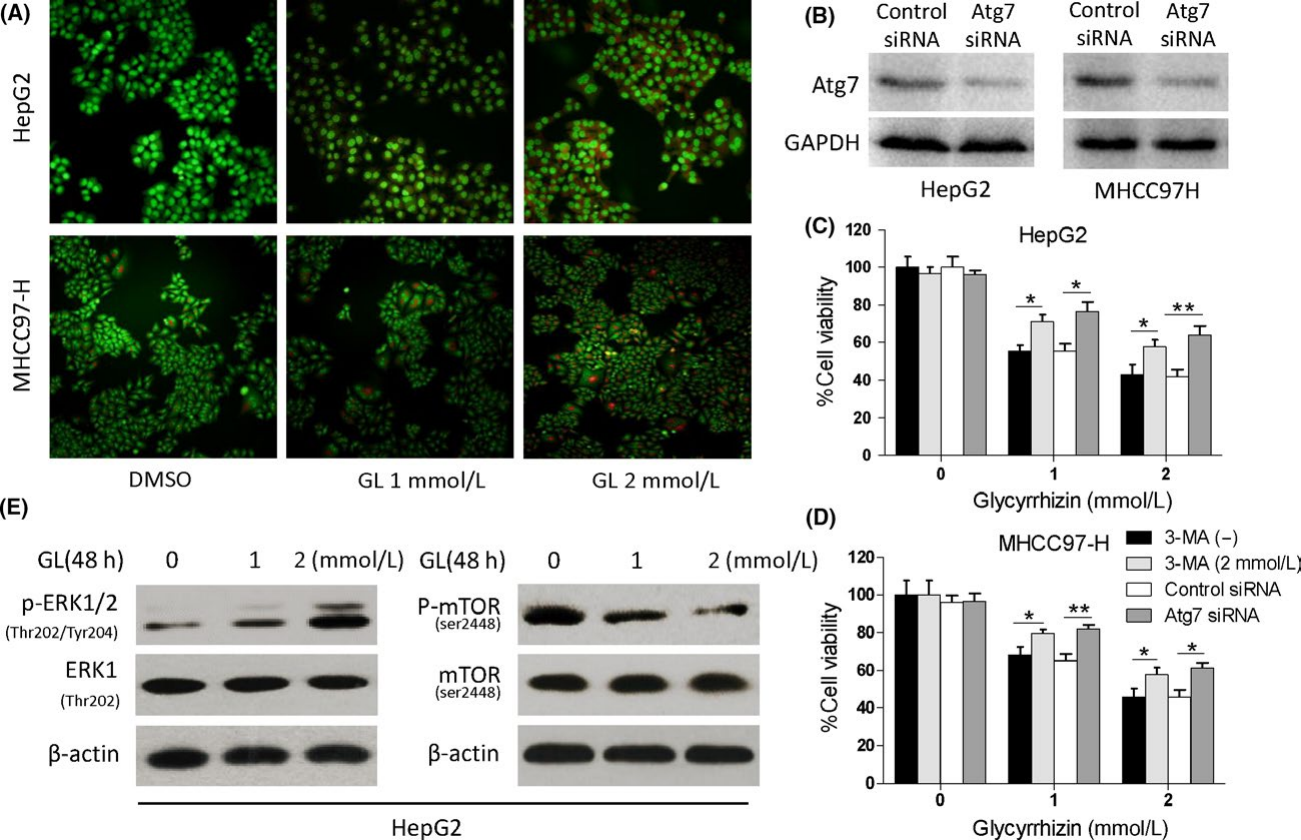
Glycyrrhizin increased the level of autophagy in HCC cells via inhibiting mTOR signaling and enhancing ERK1/2 activity. (A) HepG2 and MHCC97‐H cells were exposed to the indicated concentrations of glycyrrhizin (0, 1, or 2 mmol/L) for 12 h. The autophagic efficacy of HCC cells was determined by acridine orange staining, and the formation of acid vesicular organelles was evaluated by fluorescence microscopy. Representative images of cell autophagy in HepG2 and MHCC97‐H cells were taken after 12 h treatment with or without glycyrrhizin under a 200× microscope field. (B) The efficiency of Atg7 knockdown. HepG2 and MHCC97‐H cells treated with Atg7 siRNA transfection showed lower protein expression of Atg7 as confirmed by western blotting. (C) and (D) The effect of autophagy inhibition on the cytotoxicity of GL. After HepG2 and MHCC97‐H cells were treated with 0, 1, or 2 mmol/L GL with or without 2 mmol/L 3‐MA and 50 nmol/L Atg7 siRNA pretreatment for 48 h, the cell viability was measured by a CCK‐8 assay. The viability of untreated cells was considered 100%. (E) Detection of p‐ERK1/2 and p‐mTOR proteins in HepG2 cells by western blot analysis after exposure to glycyrrhizin for 48 h. Columns, mean (*n* = 3, in triplicate); bars, SD. *P < 0.05; **P < 0.01.

**Figure 4:**
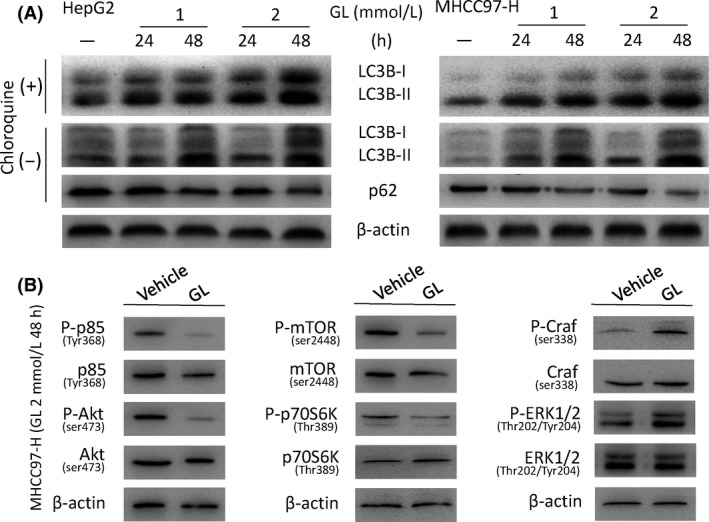
Glycyrrhizin‐induced generation of LC3B is relevant to the regulation of the PI3K/Akt/mTOR and ERK1/2 pathways. (A) Glycyrrhizin evoked the expression of LC3B in the presence and absence of chloroquine accompanied by the decline of p62 in a dose‐ and time‐dependent manner. HepG2 and MHCC97‐H cells were exposed to glycyrrhizin at various doses for various periods of time. Total cell lysates were subjected to LC3 and p62 protein analysis by western blot. *β*‐Actin was used as a loading control. (B) Glycyrrhizin inhibited PI3K/Akt/mTOR signaling and enhanced the activity of the ERK1/2 pathway. MHCC97‐H cells were treated with 2 mmol/L glycyrrhizin for 48 h. Total cell lysates were analyzed by western blot to determine the level of p‐p85, p‐Akt, p‐mTOR, p‐p70S6K, p‐Craf, and p‐ERK1/2 protein expression.

To investigate whether autophagy played a role in the GL‐induced cytotoxicity efficacy in HCC cells, we detected the GL‐induced autophagic response in HepG2 and MHCC97‐H cells. AO is usually used as a fluorescent molecule to identify either apoptotic or autophagic cell death. It can interact with DNA and emit green fluorescence or accumulate in acidic organelles in which it becomes protonated and forms aggregates that emit bright orange/red fluorescence. AO staining showed that acidic autophagic vacuoles, which emit bright orange/red fluorescence, obviously increased both in HepG2 and MHCC97‐H cells administered with different dosages (1 or 2 mmol/L) of GL for 12 h compared with untreated groups, and this increase was in a dose‐dependent manner (Fig. 3A). In addition, an autophagosome‐specific protein, LC3, associated with the membrane of autophagosomes was examined with western blot to further assess the induction of autophagy. Autophagic flux was verified by chloroquine administration and the degradation of selective autophagic targets, SQSTM1/p62. The expression of LC3‐II increased in HepG2 and MHCC97‐H cells accompanied by the decline of p62 exposed to GL in dose‐ and time‐dependent manners (Fig. 4A). Both these data indicate that GL induces autophagy in HepG2 and MHCC97‐H cells. Whether GL mediated autophagy is a protective response or an anticancer toxicity is still unclear. In order to assess the positive or negative efficacy of GL‐induced autophagy, we tested whether inhibition of autophagy affects the cytotoxicity of GL. When GL‐induced autophagy was inhibited by 3‐MA and Atg7 siRNA (Fig. 3C and D), the declined survival of HepG2 and MHCC97‐H cells administered with GL was reversed. These data suggest that GL‐induced autophagy is an excess autophagy which can result in cell death and exhibit an antitumor effect.

### Glycyrrhizin inhibits Akt/mTOR signaling and activates ERK1/2 in HCC cells

Because the Akt/mTOR pathway is one of the main regulatory pathways which negatively regulates autophagy, we investigated the effect of GL on this pathway using western blotting. Administration of GL decreased phosphorylated p85 and Akt effectively in MHCC97‐H cells (Fig. 4B). These data prompt that GL influenced the upstream pathway of Akt in induction of autophagy. Administered with GL also both decreased phosphorylated mTOR in HepG2 (Fig. 3E) and MHCC97‐H cells, accompanied by reduced phosphorylated p70S6K (Fig. 4B). Because the ERK1/2 pathway positively regulates autophagy in cancer cells during starvation, we also examined this pathway after GL treatment. GL exposure increased phosphorylated ERK1/2 in HepG2 and MHCC97‐H cells (Figs. 3E and 4B). These data indicate that GL can silence the Akt/mTOR pathway and enhance the ERK1/2 activity in induction of excess autophagic cell death.

### Glycyrrhizin suppresses in vivo growth of tumor xenografts via autophagy

**Figure 5:**
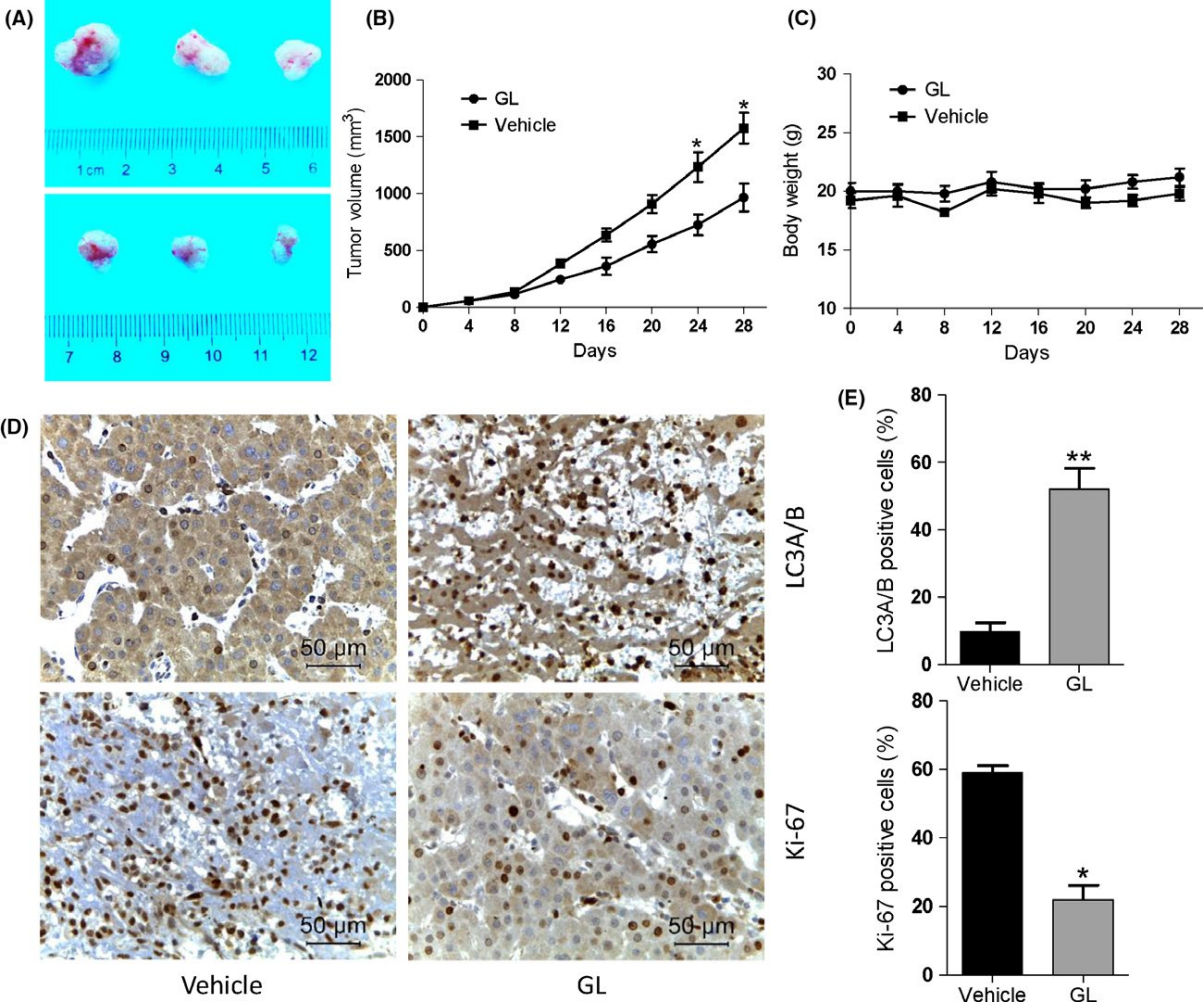
Glycyrrhizin suppresses in vivo tumor growth in a xenograft model. (A) Nude mice bearing MHCC97‐H cells were treated daily with the vehicle or glycyrrhizin at 100 mg/kg body weight by peritumoral administration for 40 days. Representative mice with MHCC97‐H xenografts and tumor masses are shown. (B) It was found that treatment with glycyrrhizin obviously suppressed tumor volumes compared to the vehicle control group, indicating that glycyrrhizin could significantly inhibit the MHCC97‐H tumor growth in vivo. Curves, mean (*n* = 3, in triplicate). (C) Glycyrrhizin resulted in little toxicity effects in vivo. No significant differences in body weights were detected among all the groups. (D) Histological analysis of the tumors from (A) and (B) was performed by examining Ki‐67 and LC3B expression to compare cell proliferation and autophagy in glycyrrhizin‐ and vehicle‐treated tumors. Scale bars, 50 *μ*m. (E) Quantification of Ki‐67‐ and LC3B‐positive cells in (D). Values are the means ± SD of triplicate samples.*P < 0.05; **P < 0.01.

To further assess the antitumor efficacy of glycyrrhizin in vivo, we executed subcutaneous tumor xenotransplant experiment. Nude mice bearing MHCC97‐H HCC cell xenografts were administered with glycyrrhizin by peritumoral injection once per day and the tumor growth rate was monitored and calculated. The results showed that glycyrrhizin obviously inhibited the growth of HCC tumor grafts compared with the control group. Calculated tumor volumes in the glycyrrhizin‐administered group were 39 ± 8% of those in the control group (Fig. 5A and B). In addition, glycyrrhizin treatment did not exerted apparent signs of toxicity or leaded to any loss of body weight in nude mice during the experiment (Fig. 5C), demonstrating that glycyrrhizin can be generally tolerated in vivo. Furthermore, we investigated the effect of glycyrrhizin on cell autophagy and proliferation in vivo by examining LC3B and Ki‐67 expression in tumor tissues harvested from vehicle‐ or glycyrrhizin‐treated mice. Glycyrrhizin administration resulted in a 3.1‐fold decline in Ki‐67‐positive cells in tumor tissue sections harvested from glycyrrhizin‐administered mice compared to vehicle‐treated mice (Fig. 5D and E, lower). Meanwhile, a 4.3‐fold increase of LC3B‐positive cells was found in glycyrrhizin‐treated tumor sections relative to vehicle‐treated groups (Fig. 5D and E, upper). As a result, glycyrrhizin is proved to be potent in inhibiting the growth of MHCC97‐H cell‐derived HCC tumors in vivo.

## Discussion

Glycyrrhizin (GL), a triterpenoid saponin from *Glycyrrhiza glabra* roots (licorice), exhibited various pharmacological effects 19, 26. GL was recently demonstrated to induce apoptosis and showed an anticancer ability in many types of cells, such as human endometrial cancer cells, leukemia cells 13, and a glioblastoma cell line 6. GL also potently inhibited the growth of breast cancer stem/progenitor cells 27. In our study, GL exhibited a significant cytotoxic effect on HCC cell lines with dose‐ and time‐dependent manner. This is consistent with other scientists' researches. Cell proliferation and migration are closely related to cancer progression and play an important role in the process of HCC; therefore, we examined whether GL showed antiproliferative and antimigration effects on HCC cells. The results showed that GL markedly inhibited HepG2 and MHCC97‐H cell proliferation in concurrence with effective inhibition of HepG2 and MHCC97‐H cell migration.

It is undeniable that GL exhibited its anticancer role partly through inducing apoptosis in cancer cells. In addition to apoptosis, many studies have recently focused on anticancer drug‐induced nonapoptotic cell death, such as necroptosis and autophagic cell death 28, 29. Laconi found that triterpene glycyrrhizin was a strong inducer of autophagy and demonstrated its ability to induce the autophagic process activator Beclin1 in epithelial cells 30. Treatment with 70% ethanol extracts of 228 *μ*g/mL licorice for 24 h in LNCaP prostate adenocarcinoma cells induced autophagy‐related cell death by downregulating the Bcl‐2 protein and inhibiting the mTOR pathway 31. Our group also investigated the impact of GL on HCC cells' autophagic phenomenon by AO staining and LC3B detection. Interestingly, we found that HCC cells exposed to GL had an obvious autophagic phenomenon compared to the control group. The degree of autophagic response was correlated with the concentration of GL. This result supports the hypothesis that GL may exhibit its role of cytotoxicity in HCC cells partly through excess autophagy induction.

Ordinarily, autophagic phenomenon can be triggered by growth factor depletion, oxygen and energy deficiency, or the upregulation of Bcl‐2 protein, etc. It was originally considered to be a strategy to accelerate material and energy recycling via digestion of aged organelles with autophagy‐specific lysosomes, which was beneficial for cell proliferation under stress or in some nutrient‐insufficient microenvironments 28, 32. The prosurvival efficacy of autophagy in cancer had long been speculated and was deemed reasonable. However, in recent years, increasing evidences manifested that autophagy might also play an important role in tumor suppression, and it had been designated as “programmed cell death type II.” The early upregulation of autophagy exerted cytoprotective activity, but further upregulation increased autophagy‐induced cell death 33. During the entire process of autophagy, the cytoplasm and organelles are damaged, which is represented by damaged mitochondria and endoplasmic reticulum. Although autophagy does not directly damage the cell membranes or nucleus, there is evidence that after an initial fracture or digestion, the cell membrane and nucleus will eventually become lysosomes to digest and break down their own structures.

Even the definition of autophagic cell death are still under debate. Increasing studies strongly support its role in tumor suppression. Gurpinar et al. 34 demonstrated the involvement of autophagic cell death in lung adenocarcinoma cells induced by sulindac sulfide amide treatment. Intriguingly, cell death occurred in this system was independent of caspase activation 34. Meanwhile, Lamy et al. 35 proved that autophagic activity in myeloma cells was restricted to the cleavage of an autophagic inducer, BCL2 interacting protein BCLAF1 and then cell death could be avoided. Furthermore, SI113, worked as an SGK1 inhibitor, could also induce cytotoxic autophagy in human glioblastoma multiforme cells 36. Although autophagy‐mediated cell death is just one mechanism of cell death, it still evokes a keen interest in scientists.

Recognizing that autophagy is a double‐edged sword, whether autophagy actually causes hepatocellular carcinoma cells to death or exerts a protective efficacy has been a controversial issue. In our research, we explored the anticancer efficacy of GL on HepG2 and MHCC97‐H human HCC cells in vitro and in vivo. We surprisedly found that GL‐associated cytotoxicity in HCC was partly mediated by autophagic cell death. Furthermore, we investigated the signal pathways involved in GL‐induced autophagy and their roles in resulting in cell death. To the best of our knowledge, this is the first study to demonstrate that GL can provocate autophagic phenomenon to cause excessive self‐digestion in HCC cells in vitro and in vivo. This disorder of self‐destruction may be regulated by simultaneous inhibition of Akt/mTOR pathway and stimulation in ERK1/2 activity. This result further contributes to the verdict that autophagy‐mediated cell death is also a credible route of tumor suppression pathway 37.

According to previous studies on the molecular mechanism of autophagy, various signal pathways are involved in the progress, including PI3K/Akt/mTOR, ERK1/2, class I PI3K/PKB, G*α*i_3_ protein, NF‐*κ*B, and calcium pathways. Our data manifested that GL seemingly could restrain the Akt/mTOR pathway and activate ERK1/2 activity to lead to excess induction of autophagy in HCC cells (Fig. 4B). These two pathways differently influenced the autophagy and contributed to the cytotoxicity of GL. The mTOR and ERK1/2 pathways both importantly participated in the process of autophagy occurred in various cancer cell types. The PI3K/Akt/mTOR pathway was inhibited by an andrographolide analog and caused apoptosis and autophagic cell death in U937 cells 38. Naringin was found to induce autophagic growth inhibition via downregulation the PI3K/Akt/mTOR cascade in concurrence with activation of MAPK pathways in AGS cancer cells 39. Resveratrol‐activated autophagy mediated cell death in prostate cancer cells via downregulating of STIM1 and mTOR pathway 40. Raphael found that GL had a marked inhibitory role in AKT/mTOR signaling and resulted in repressed growth of breast cancer stem/progenitor cells 27. Some scientists also investigated the effect of GL on ERK signaling, but with different results. Teng et al. 41 showed that GL markedly upregulated the expression of phosphorylated extracellular signal‐regulated kinase (p‐ERK) and mediated its migration from cytoplasm to nucleus in dopaminergic neuronal cells. In contrast, Tu et al. 42 found that GL significantly repressed the concanavalin A‐induced phosphorylation of ERK in vitro.

The detailed modulatory mechanism mediated by GL in different pathways might vary due to the cell types. The mTOR inhibition in concurrence with ERK activation might be one of the common mechanisms in autophagy induction. Ellington et al. 43 showed the similar data in accordance with our findings. Inhibited Akt signaling and enhanced ERK activity were both simultaneously observed in the process of autophagy induction by natural products triterpenoid B‐group soyasaponins. 3‐Methyladenine (3‐MA) is a nonselective PI3K inhibitor which can both combine with Vps34 and PI3Ki to block *β* and I type PI3K. The inhibitory of PI3K*γ* may contribute to the block of I type PI3K by GL. The role of autophagy in GL‐induced cell death was also confirmed by knocking down autophagy‐essential gene *Atg7*. Meanwhile, the role of ERK in autophagy induction should also be confirmed by genetic approaches and these need further investigation in the future.

Considering the dose‐ and time‐dependent manner, we concluded that autophagy could be evoked by GL in HepG2 and MHCC97‐H cells. Furthermore, GL significantly inhibited tumor growth accompanied by autophagy occurred actively in the xenograft tumor model of MHCC97‐H cells. Our data clearly manifest a fact that GL can trigger excessive autophagic phenomenon and cause the metabolic disorder in HCC cells which finally result in autophagy‐mediated cell death and exerting a cytotoxic efficacy. These results indicate that GL might be a promising agent for clinical application in patients with HCC.

## Conflict of Interest

All the authors declared no competing interests.
